# Associations between self-reported masticatory dysfunction and frailty: A systematic review and meta-analysis

**DOI:** 10.1371/journal.pone.0273812

**Published:** 2022-09-09

**Authors:** Gotaro Kojima, Yu Taniguchi, Masanori Iwasaki, Reijiro Aoyama, Tomohiko Urano

**Affiliations:** 1 Department of Research, Dr. AGA Clinic, Tokyo, Japan; 2 Center for Health and Environmental Risk Research, National Institute for Environmental Studies, Tsukuba, Japan; 3 Tokyo Metropolitan Institute of Gerontology, Tokyo, Japan; 4 Department of Japanese Studies, The Chinese University of Hong Kong, Shatin, Hong Kong; 5 Department of Geriatric Medicine, International University of Health and Welfare, Chiba, Japan; Ritsumeikan University, JAPAN

## Abstract

**Background:**

Oral health is a key factor of overall health and closely associated with well-being and quality of life. Mastication is one the most important oral functions and may deteriorate with aging. Evidence on association between masticatory dysfunction and frailty in the literature is scarce and not coherent.

**Methods:**

A search strategy was developed to conduct a systematic review of the literature in PubMed, CINAHL, and AMED in accordance with the PRISMA 2020 guidelines. We searched for studies published in 2000 or later that examined associations between self-reported masticatory dysfunction and frailty risk. The reference lists of the relevant articles were reviewed for additional studies. We calculated pooled odds ratios (OR) of association between self-reported masticatory dysfunction and the risk of frailty by fixed-effects meta-analysis. The Joanna Briggs Institute Critical Appraisal Checklist was used to assess risk of bias. Publication bias was assessed by visually inspecting a funnel plot.

**Results:**

A total of 285 studies were identified by the literature search. Among 5 studies selected for this review, 4 cross-sectional studies including a total of 7425 individuals were used for meta-analysis. The pooled results by a fixed-effects model showed that there was a significant association between self-reported masticatory dysfunction and frailty risk (pooled OR = 1.83, 95%CI = 1.55–2.18, p<0.00001). There was no evidence of publication bias observed.

**Conclusions:**

This systematic review and meta-analysis highlighted pooled cross-sectional evidence that community-dwelling older people who report masticatory dysfunction are significantly more likely to be frail than those who do not. The limitations of this study are: inclusion of only cross-sectional studies, no gold standard to measure masticatory functions, self-reported information on masticatory function, and the limited number of included studies. More longitudinal studies are warranted for further understanding of the causal pathways and elucidate underlying mechanisms.

Registration: PROSPERO CRD42021277173

## Introduction

Oral health is a key factor of overall health and closely associated with well-being and quality of life [1]. Good oral health plays an important role regarding nutrition, employment, self-esteem, and social interaction, and is imperative for healthy aging [2]. The World Health Organization has identified global health challenges posed by oral diseases and been committed to achieving better oral health [2].

Physical frailty has now been widely accepted as an important health issue and is being getting integrated into clinical practice and guidelines [3, 4]. Recently, a new concept, “oral frailty”, has been proposed as a series of processes leading to an age-related decline in oral conditions associated with increased vulnerability to oral dysfunction as well as physical and psychological health deterioration [5]. Previous studies have shown that oral frailty is associated with poor physical function, physical frailty, sarcopenia, disability, and premature death [6–8]. Its associations with worse social and nutritional profile were also observed [9, 10].

Along with impaired oral functions and conditions associated with oral frailty, such as loss of teeth, poor oral hygiene, or dysphagia, masticatory dysfunction is particularly relevant to oral health [11, 12]. Mastication, or chewing, is one the most important oral functions. It consists of multifaceted processes, involving multiple oral structures and muscles working in harmony, to break down food into smaller particles and permit swallowing. Masticatory dysfunction refers to a debilitating condition where normal masticatory function is compromised due to either structural or functional factors [13]. It is common that masticatory function is affected with aging and the prevalence of masticatory dysfunction is high in older adults [12, 14]. Maintaining or restoring proper mastication can be considered as the most important objective of dental care [15].

Evidence on association between masticatory dysfunction and frailty in the literature is mixed: some studies have documented significant associations [16], but others did not [17, 18]. Although several review papers have investigated frailty and oral health indicators, including masticatory dysfunction [16, 19–21], none of them specifically focused on masticatory dysfunction or conducted a meta-analysis. Therefore, the objectives of this study are to perform a systematic literature review specifically on the association between self-reported masticatory dysfunction and frailty among community-dwelling older adults, and to synthesize pooled quantitative evidence by conducting a meta-analysis. The research question based on the PICO format considered whether community-dwelling older adults (P) with self-reported masticatory dysfunction (I), compared with those without (C), were more likely to have a higher risk of frailty (O). This study specifically focused on self-reported masticatory dysfunction as different devices and definitions were used for objectively measured masticatory dysfunction, without a gold standard.

## Methods

### Search strategy and study selection

The protocol of the literature search was generated according to the Preferred Reporting Items for Systematic Review and Meta-Analyses (PRISMA) 2020 [22] and registered at PROSPERO (http://www.crd.york.ac.uk/PROSPERO/display_record.php?ID=CRD42021277173). Three electronic databases (PubMed, CINAHL, and AMED) were searched on 12 September 2021 for studies published in 2000 or later that examined association between self-reported masticatory dysfunction and frailty risk. An explosion function was used if available, and no language restriction was applied. The search strategy used a combination of Medical Subject Heading (MeSH) terms and text terms as followed: (Masticatory muscles (MeSH) OR Bite force (MeSH) OR Mastication (MeSH) OR masticat* OR chew* OR occlu*) AND (Frailty (MeSH) OR frailty OR Frail elderly (MeSH)). Reference lists of included studies and relevant studies were scrutinized manually.

All cross-sectional or prospective studies that examined associations between self-reported masticatory dysfunction and frailty risk among community-dwelling older adults with a mean age of 60 years or greater and provided effect measures, such as odds ratio (OR) or hazard ratio (HR), were eligible. Self-reported masticatory dysfunction was considered to be present when a participant subjectively reported difficulty or impairment in chewing, biting, eating hard foods, or food in general. Frailty should be defined using validated criteria, such as the frailty phenotype [23] or the Frailty Index [24]. Studies were excluded if they were randomized controlled trials, reviews, editorials, letters, comments, or conference abstracts. When the same cohort was used by multiple studies, the one with the largest sample size was selected. An adjusted effect measures were preferred when adjusted and unadjusted ones were presented. Three researchers (GK, YT, and MI) performed title, abstract, and full-text screening independently for eligibility. Disagreement was resolved by discussion.

### Data extraction

The characteristics and findings extracted from the included studies by GK were first author, publication year, study cohort name, location, sample size, proportion of female participants, age (mean and range or age criterion for inclusion), frailty criteria used, study design (cross-sectional vs. longitudinal), follow-up period if prospective, and findings of interest.

### Methodological quality assessment

Studies considered to be eligible based on screening of title, abstract, and full-text were examined by GK for methodological quality using the 8-item Joanna Briggs Institute Critical Appraisal Checklist for Analytical Cross-Sectional Studies [25]. If a study met 4 or more criteria, it was considered to have adequate quality of methodology.

### Statistical analysis

A meta-analysis was attempted if two or more studies provided the same effect measures regarding associations between self-reported masticatory dysfunction and frailty. The presence of heterogeneity was assessed using the chi square test and was considered to be present if the p value was less than 0.05. The degree of the heterogeneity was assessed using I^2^ statistic. When heterogeneity was found to be present, a random-effects model was used, while a fixed-effect model was used instead when the heterogeneity was absent. If heterogeneity was identified, subgroup and sensitivity analyses were considered to explore possible causes of heterogeneity. Publication bias was examined by visually inspecting a funnel plot.

All analyses were performed using Review Manager 5 (version 5.2, The Cochrane Collaboration, Copenhagen, Denmark), and the level of significance was set at *p* ≤0.05.

## Results

### Selection processes

**Fig 1:**
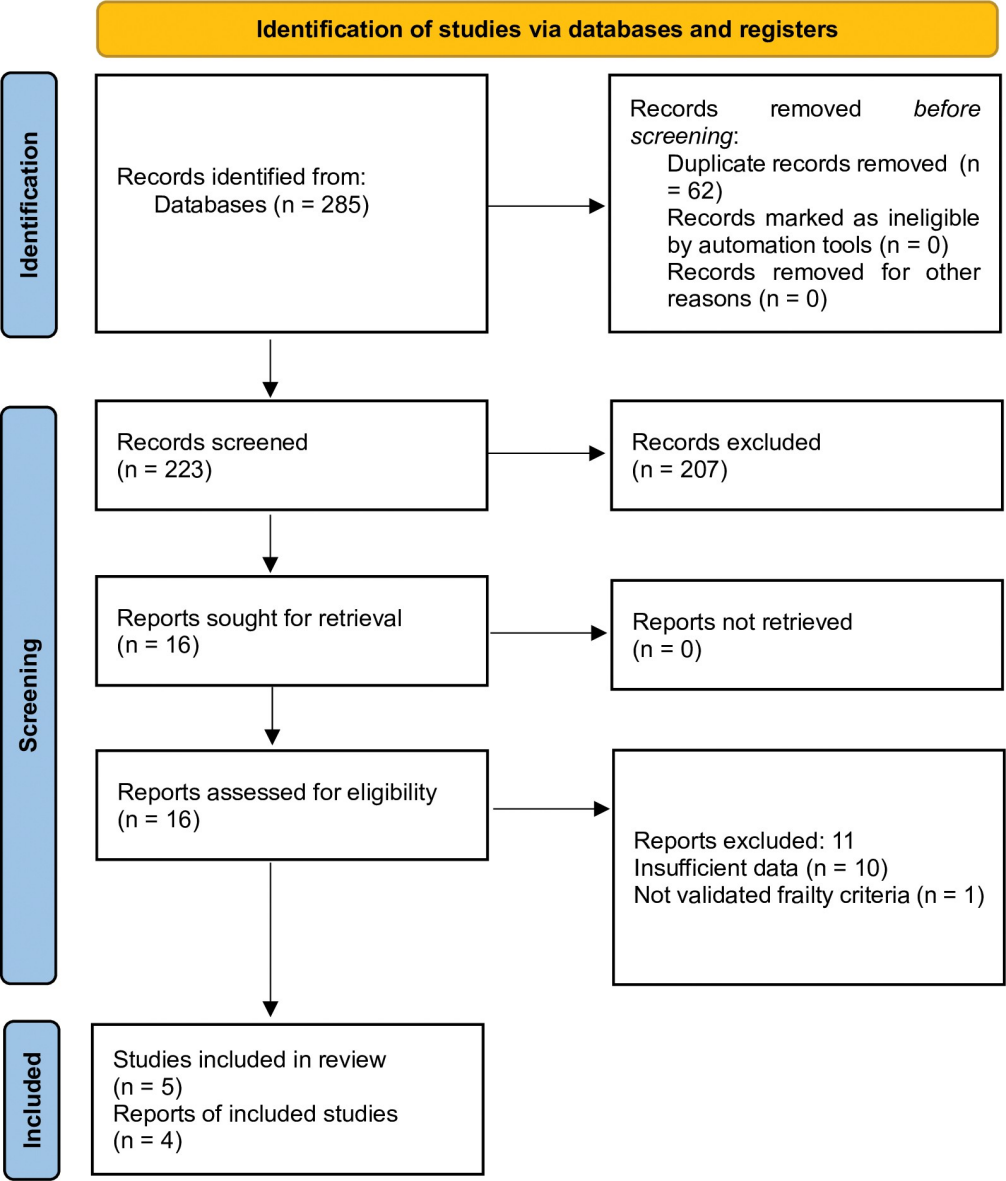
Flow chart of systematic literature review.

A systematic literature search using three databases yielded a total of 285 studies (214 from PubMed, 65 from CINAHL, and 6 from AMED). After 62 duplicates were removed, 223 studies were screened for title and abstract, by which 207 studies were excluded. Sixteen studies were left for full-text assessment, among which 11 studies were found to not provide relevant and/or sufficient data to be included. Two studies used the Kihon Checklist (KCL) to define frailty and examined cross-sectional associations [26] and risk of worsening frailty status by baseline masticatory dysfunction [27], respectively. Although KCL is a validated tool to measure frailty [28], one of the KCL criteria is “difficulty eating tough foods” and it was considered to be reasonable to exclude these studies for this review. Finally, the remaining 5 cross-sectional studies [12, 17, 18, 29, 30] were considered to meet the inclusion criteria and included for this review. A PRISMA flow chart is shown in **Fig 1**.

### Study characteristics

A summary of the study characteristics and findings is presented in **Table 1**. Most studies (4 out of 5) have been published recently, in 2018 or later [12, 17, 18, 30], and one study was published in 2012 [29]. Included studies were from Japan [17], China [30], Hong Kong [12], Thailand [18], and Mexico [29]. The sample sizes of the cohorts were from 141 to 3635, and the proportion of female participants were 51.8% to 76.9%. Four studies provided mean age of the cohorts, which ranged from 70s to 80s. The frailty definitions used were the Fried’s phenotype criteria [17, 18, 29], the Frailty Index [30], and FRAIL scale [12]. All studies were examined for methodological quality and considered to have adequate quality (score range = 6–8, mean = 7.5). Of the 5 studies included, 4 studies [12, 17, 29, 30] provided ORs from binary logistic regression models, while the other [18] used an ordinal logistic regression model. A significant association between self-reported masticatory dysfunction and frailty was observed in 3 studies, and two other studies did not reach statistical significance. No study showed significant inverse associations between self-reported masticatory dysfunction and frailty.

**Table 1:** Summary of included studies examining associations between self-reported masticatory dysfunction and frailty risk*.

Author/Year/Study/Title	Location	Sample size	Female (%)	Age (range)	Frailty criteria	Findings
Nakamura 2021 [17]	Japan	832	63.6%	74.9	mCHS	- aOR = 1.52 (0.73–3.16) of subjectively reported decreased masticatory function for frailty compared with non-frailty.
2018 Tarumizu Study				(≥65)		
Gu 2019 [30]	China	3635	51.8%	84.3	FI>0.21	- aOR = 1.64 (1.28–2.08) of reporting pain on chewing for frailty compared with non-frailty.
Chinese Longitudinal Healthy Longevity Survey				(≥65)		
Iwasaki 2018 [18]	Thailand	141	71.6%	72	mFF	- uOR = 1.10 (0.51–2.35) of self-perceived difficulty with chewing for severity of frailty status by ordinal logistic regression model.
				(≥60)		
Woo 2018 [12]	Hong Kong	2259	76.9%	-	FRAIL scale	- aOR = 1.53 (1.22–1.91) of self-reported chewing difficulty for prefrailty compared with robust.
				(≥60)		
						- aOR = 2.21 (1.61–3.04) for frailty compared with robust.
Castrejon-Perez 2012 [29]	Mexico	699	53.2%	77.9	mFF	- uOR = 1.97 (1.29–3.00) of self-reported chewing problems for frailty compared with non-frailty
Mexican Study of Nutritional and Psychosocial Markers of Frailty				(≥70)		

* All cross-sectional studies.

aHR: Adjusted hazard ratio

aOR: Adjusted odds ratio

C: Cross-sectional study design

FI: Frailty Index

L: Longitudinal study design

mFF: Modified Fried’s phenotype criteria

OSHPE: Obu Study of Health Promotion for the Elderly

uOR = Unadjusted odds ratio

### Meta-analysis of cross-sectional association between self-reported masticatory dysfunction and frailty

**Fig 2:**
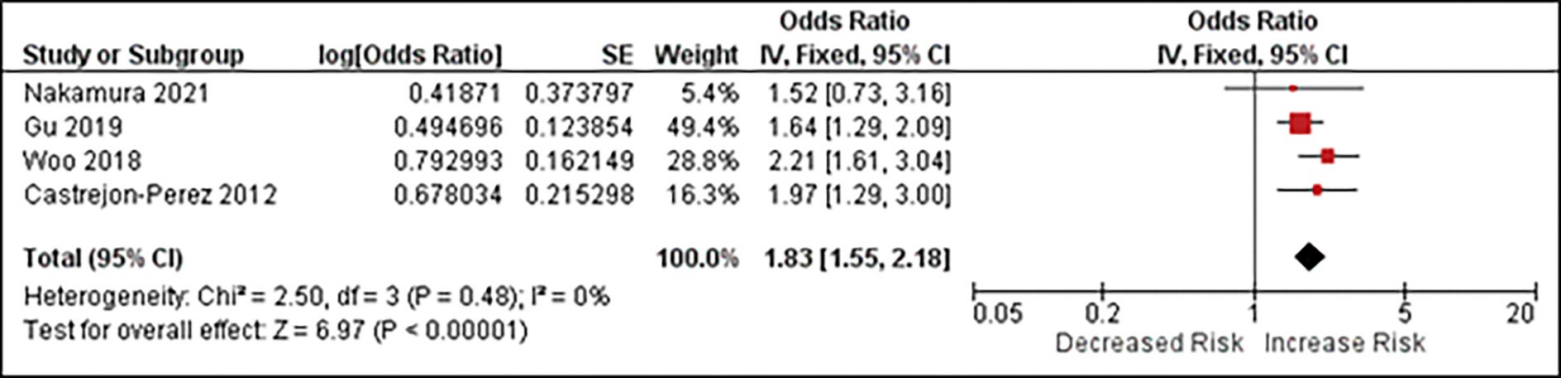
Forest plot of cross-sectional association between impaired self-reported masticatory dysfunction and prevalent frailty risk.

ORs from 4 studies [12, 17, 29, 30] involving 7425 individuals were combined to calculate pooled estimate regarding association between self-reported masticatory dysfunction and frailty. A fixed-effects model was chosen due to absence of heterogeneity (p = 0.48, I^2^ = 0%). The synthesized results showed that there was a significant association between self-reported masticatory dysfunction and frailty risk (pooled OR = 1.83, 95%CI = 1.55–2.18, p<0.00001). (**Fig 2**)

### Publication bias assessment

Publication bias was assessed across 4 studies. No evidence of publication bias was observed by visually inspecting a funnel plot.

## Discussion

A systematic review of the literature was conducted for currently available evidence on the association between self-reported masticatory dysfunction and frailty among community-dwelling older adults and identified 5 studies. Four of them were used for meta-analysis, showing the significant association between self-reported masticatory dysfunction and frailty.

Masticatory function is commonly affected in older adults. Factors associated with impaired masticatory function are loss of teeth, ill-fitting denture, decreased saliva flow, weakened masticatory muscles, and tooth or jaw pain [12, 15, 31]. Masticatory function seems to be closely and complexly affected by other oral factors. For example, salivary flow rate was significantly correlated with masticatory performance only in those who had no occlusal contact but not in those with some contact [15]. In addition, the number of residual teeth was significantly associated with masticatory performance only in those with some contacts but not in those without [15]. Effect measures provided by the included studies were not adjusted or stratified for these factors, which thus may have yielded residual confounding.

It is not known how masticatory dysfunction and frailty are related. As the data used by the current meta-analysis were all cross-sectional, causal relationships cannot be inferred from the pooled findings. However, there seem to be plausible biological theories supporting bidirectional relationship. It is possible that masticatory dysfunction can lead to frailty through malnutrition [32]. It has been revealed that masticatory dysfunction may be responsible for older people’s changing their food orientation. Those with masticatory dysfunction tend to consume predominantly soft and easy to chew foods, and their nutritional intake may be insufficient [33, 34]. On the other hand, frailty can possibly predispose to masticatory dysfunction. Sarcopenia, a core feature of frailty, is an age-related decrease in muscle mass and function and can affect all muscle of the body [35]. Therefore, older adults with frailty may have impaired masticatory muscles, leading to a complaint of difficulty in chewing. Very few longitudinal studies examined oral health and subsequent frailty risks. Among them, a Japanese study showed that difficulties eating tough foods were reported more in older adults who later developed frailty compared with those who did not (26% vs. 16%) [7].

There are some limitations that should be noted. First, as the current meta-analysis included only cross-sectional studies, causal relationships or the direction of the pathway between masticatory dysfunction and frailty cannot be inferred. Second, there are several methods to evaluate masticatory functions but without a gold standard [36]. It is also difficult to measure masticatory dysfunction objectively. The information used for the current review were all self-reported; therefore, self-report bias is inevitable. Third, the included studies used different frailty definitions, while the findings are mostly consistent and the heterogeneity was quite low. Lastly, due to the limited number of the included studies, subgroup analysis or sensitivity analysis were not performed.

Despite these limitations, this study has significant strengths. First, the current study was conducted based on the predefined protocol developed according to the PRISMA guidelines, using multiple databases. The search strategy was comprehensive and reproducible, using a combination of MeSH and text terms. Screening was done by three researchers independently and the potentially eligible studies were assessed for methodological quality. Second, most of the effect measures from the included studies were adjusted for potential confounders, and the meta-analysis showed low heterogeneity (I^2^ = 0%).

### Conclusions

This systematic review and meta-analysis highlighted pooled cross-sectional evidence that community-dwelling older people who report masticatory dysfunction are significantly more likely to be frail than those who do not. More longitudinal studies are warranted to enhance our understanding of the causal pathways and to elucidate underlying mechanisms, which may help develop countermeasures to address masticatory dysfunction, frailty, or both.

## Supporting information

S1 Checklist (DOCX)

S1 Dataset (XLSX)

## Abbreviations

HR: Hazard ratio
KCL: Kihon Checklist
MeSH: Medical Subject Heading
OR: Odds ratio; PRISMA
Preferred: Reporting Items for Systematic Review and Meta-Analyses
